# The Hidden Dangers of Hypercalcemia: A Case Report of Undiagnosed Symptoms Leading to Complications

**DOI:** 10.7759/cureus.78631

**Published:** 2025-02-06

**Authors:** Ana Filipa Silva, Francisco Belchior, Francisco Pombo, Lindora Pires

**Affiliations:** 1 Internal Medicine, Centro Hospitalar Tâmega e Sousa, Penafiel, PRT; 2 Internal Medicine, Centro Hospitalar Tamega e Sousa, Penafiel, PRT

**Keywords:** calcium homeostasis, generalized tonic-clonic seizures, hypercalcemia, primary hyperparathyroidism, zoledronic acid

## Abstract

Hypercalcemia is a relatively common metabolic abnormality; however, it is frequently undiagnosed. It often manifests with neuropsychiatric, gastrointestinal, renal, and musculoskeletal symptoms. We report the case of a 75-year-old woman with a history of syncope, anorexia, asthenia, weight loss, polydipsia, polyuria, visual hallucinations, confusion, generalized tonic-clonic seizures, and depression. Laboratory workup revealed severe hypercalcemia (ionized calcium: 1.81 mmol/L) with normal renal function and significantly elevated parathyroid hormone (PTH) levels. A diagnosis of primary hyperparathyroidism was made, and the patient was treated with fluid therapy and zoledronic acid, resulting in the resolution of the symptoms.

## Introduction

Hypercalcemia is a relatively common metabolic disturbance that often remains undiagnosed, with a prevalence of approximately 1-2% in the general population. It is characterized by elevated calcium levels in the blood and can be caused by various conditions [1,2] including primary hyperparathyroidism, malignancy, vitamin D excess, kidney disease, medications, dehydration [3], and granulomatous diseases. 

In malignancies, hypercalcemia can arise due to humoral factors secreted by tumors, which disrupt normal calcium homeostasis. The PTHrP (parathyroid hormone-related protein) plays an important role in hypercalcemia of malignancy. PTHrP is a protein produced by several types of malignant tumors, such as those in the breast, lungs, kidneys, and others. It mimics the action of parathyroid hormone (PTH), which regulates calcium metabolism, but in a dysfunctional way. 

PTHrP can bind to PTH receptors in bones, stimulating the activity of cells called osteoclasts. These osteoclasts are responsible for bone resorption or the release of calcium from bones into the bloodstream. This process raises calcium levels in the blood.

PTHrP can also act on the kidneys, promoting calcium reabsorption in the renal tubules, further contributing to hypercalcemia. It can also interfere with the production of 1,25-dihydroxyvitamin D, an active form of vitamin D that increases intestinal calcium absorption. In some cases, PTHrP may not be directly involved in regulating 1,25-dihydroxyvitamin D levels, but its action on bones and kidneys is enough to cause hypercalcemia. Although PTHrP shares some similarities with PTH, such as the ability to increase blood calcium levels, they have important differences. PTHrP does not directly regulate the production of PTH by the parathyroid glands, and its actions are more localized, primarily in malignant tumors. PTHrP is more commonly associated with hypercalcemia in cancer patients, while PTH is more related to conditions like primary hyperparathyroidism.

Among the causes of hypercalcemia, primary hyperparathyroidism and malignancy [4] account for over 90% of cases. Generally, distinguishing between these causes is not difficult, as malignancy is often clinically apparent by the time it induces hypercalcemia. Patients with hypercalcemia due to malignancy are usually more symptomatic [5] and present with higher calcium levels than those with primary hyperparathyroidism [6].

Hypercalcemia can affect multiple organ systems, and its symptoms depend on the severity and rate of increase in serum calcium levels. The manifestations are usually nonspecific [7,8] and can range from neuropsychiatric (e.g., depression, anxiety, cognitive dysfunction) to gastrointestinal (e.g., nausea, anorexia, constipation), cardiovascular (e.g., shortened QT interval), renal (e.g., nephrolithiasis, polyuria), and musculoskeletal (e.g., muscle weakness, bone pain) symptoms. More severe hypercalcemia can lead to confusion, lethargy, stupor, and coma, especially in older patients or those with rapidly rising calcium levels [9].

## Case presentation

A 75-year-old woman with a medical history of hypertension, obesity, osteoporosis, depression, and renal lithiasis presented to the Emergency Department with episodes of syncope, significant weight loss (10 kg), anorexia, asthenia, polydipsia, and polyuria over the past six months. In the last two days, she also experienced visual hallucinations and confusion. The patient's daughter reported a history of depression, diagnosed eight months prior, which had not responded to multiple antidepressants. The physician attributed her lack of response to pathological grief after her husband's death 10 months prior. Table 1 shows the patient's laboratory results.

**Table 1:** Laboratory results

Parameter	Result at admission	Result after zoledronic acid and hydration	Reference value
Calcium	3.5 mmol/L	2.3 mmol/L	2.2-2.65 mmol/L
Albumin	4.5 g/dL	4.2 g/dL	3.5-5.2 g/dL
Corrected calcium (for albumin)	13.6 mg/dL	9.8 mg/dL	8.3-10.6 mg/dL
Ionized calcium	1.81 mmol/L	1.21 mmol/L	1-17-1.30 mmol/L
Parathyroid hormone	393 pg/mL	383 pg/mL	12-88 pg/mL
Vitamin D	31 nmol/L	31 nmol/L	>50 nmol/L
Urea	39 mg/dL	30 mg/dL	10-50 mg/dL
Creatinine	0.85 mg/dL	0.74 mg/dL	0.66-1.09 mg/dL

While in the emergency department, the patient experienced a generalized tonic-clonic seizure. On physical examination, her vital signs were as follows: blood pressure 113/68 mmHg, heart rate 70 bpm, temperature 36.3°C, and oxygen saturation 96% on 2L of supplemental oxygen. Cardiovascular and respiratory exams were unremarkable, and there was no peripheral edema.

Laboratory investigations revealed increased ionized calcium and parathyroid hormone with normal renal function and decreased vitamin D. Electrocardiogram showed sinus rhythm with a heart rate of 80 bpm, normal cardiac axis, PR interval of 140 ms, normal QRS complex, and a shortened QTc interval (454 ms). A head CT scan revealed no intracranial lesions. A chest CT angiogram showed no signs of pulmonary embolism but noted parenchymal densification at the lung bases, possibly due to aspiration.

The patient was diagnosed with primary hyperparathyroidism and started on fluid therapy with a 0.9% saline infusion until achieving adequate urine output and euvolemia, and treatment with intravenous zoledronic acid (4 mg, maximum) given as a single dose was also initiated. Both pamidronate and zoledronic acid are approved for treating hypercalcemia of malignancy; however, zoledronic acid is shown to be more effective. Zoledronic acid works by binding to the bone and inhibiting the activity of osteoclasts, the cells responsible for bone resorption. By doing so, it reduces the release of calcium from the bone matrix into the bloodstream, helping to normalize elevated calcium levels in the blood.

She was hospitalized for stabilization, clinical monitoring, and further evaluation. Respiratory failure was attributed to aspiration resulting from the generalized tonic-clonic seizure and started antibiotics with amoxicillin.

She was also evaluated by Endocrinology during the hospitalization period, starting vitamin D supplementation. At the time of discharge, she was afebrile, with no new seizures and resolution of respiratory failure. The thiazide diuretic was also discontinued as it contributed to hypercalcemia. She was referred to the Endocrinology clinic, where she is currently being monitored, having discontinued vitamin D supplementation once her vitamin D levels were normalized. No new hospitalizations have occurred since then.

## Discussion

Multiple studies have been conducted in the field of hypercalcemia, notably "Primary Hyperparathyroidism and the Risk of Cardiovascular Events: A Cohort Study," published in JAMA Internal Medicine in 2018 [10]. This study examined the association between primary hyperparathyroidism (a common cause of hypercalcemia) and the risk of cardiovascular events. The findings indicated that patients with primary hyperparathyroidism are at increased risk for both cardiac and cerebrovascular events, emphasizing the importance of early diagnosis and intervention.

Another relevant study that supports the data presented is "Hypercalcemia in Chronic Kidney Disease: Pathogenesis and Clinical Management," published in Kidney International in 2020 [11]. This article explores the mechanisms contributing to hypercalcemia in patients with chronic kidney disease, including dysfunction of the parathyroid glands and disturbances in calcium metabolism. The study also proposes therapeutic strategies for managing hypercalcemia in these patients.

Neuropsychiatric symptoms such as anxiety and depression are common. However, these symptoms can also indicate serious underlying conditions, such as hypercalcemia. Hypercalcemia can significantly affect the central nervous system (CNS), leading to a wide range of psychiatric symptoms, including confusion, delirium, depression, and, in severe cases, psychosis. The underlying mechanisms are primarily linked to the effects of elevated calcium levels on neuronal excitability and neurotransmitter function. One of the primary mechanisms by which hypercalcemia affects the CNS is through alterations in neuronal excitability since elevated serum calcium concentrations reduce the resting membrane potential of neurons, making them less excitable. This decrease in neuronal excitability can lead to disturbances in synaptic transmission and neural communication, resulting in cognitive dysfunction and altered mental status, such as confusion and disorientation. Calcium also influences the release and activity of several neurotransmitters, including dopamine, serotonin, and glutamate, which are essential for mood regulation, cognition, and behavior. Hypercalcemia can disrupt the balance of these neurotransmitters, potentially leading to psychiatric manifestations such as depression, anxiety, and psychosis. The blood-brain barrier is crucial in maintaining the homeostasis of the CNS by regulating the entry of substances into the brain. Hypercalcemia has been shown to influence the permeability of the BBB, allowing more calcium to enter the CNS and potentially exacerbating neurotoxic effects. This may lead to more severe psychiatric symptoms by directly altering brain function.

In this case, the patient's neuropsychiatric symptoms - attributed to grief after her husband's death - were initially neglected, leading to a serious and undiagnosed condition. This highlights the importance of considering organic causes in patients with psychiatric symptoms, especially when there is a lack of response to standard treatments.

The diagnosis of primary hyperparathyroidism was not initially suspected, but timely identification of hypercalcemia in the presence of neuropsychiatric symptoms led to the correct diagnosis. Clinicians should be trained to recognize the early signs of hypercalcemia. A thorough history and physical examination are vital, with particular attention to symptoms such as polyuria, polydipsia, bone pain, and gastrointestinal disturbances. The diagnosis of hypercalcemia remains challenging due to its diverse etiologies and varied clinical manifestations. However, by implementing a more structured approach to diagnosis, enhancing awareness, and utilizing advanced diagnostic technologies, clinicians can improve their ability to accurately identify the underlying causes. This will lead to more effective management and better patient outcomes, ensuring that hypercalcemia is appropriately addressed and not overlooked in clinical practice.

Notably, the patient also had evidence of target organ damage from hyperparathyroidism, including osteoporosis and renal lithiasis. Primary hyperparathyroidism remains a frequently undiagnosed condition, especially when it presents with nonspecific symptoms such as those in this case. Oftentimes, the psychiatric symptoms of hypercalcemia are mistaken for primary psychiatric disorders, such as anxiety, depression, or psychosis. Patients with hypercalcemia may experience changes in mental status, including disorientation, memory loss, and even delirium. If these symptoms are interpreted as signs of a purely psychiatric condition, the treatment for hypercalcemia may be delayed, increasing the risk of complications. Patients may be diagnosed with psychiatric disorders without proper laboratory workups to evaluate for hypercalcemia. Early recognition and treatment are essential to avoid complications.

Management strategies should be tailored to the specific cause of hypercalcemia, with a focus on both symptom control and addressing the underlying pathology. Furthermore, careful monitoring of calcium levels and related parameters is essential throughout the treatment course, as well as follow-up to detect any recurrence. Interdisciplinary collaboration, especially with endocrinologists, oncologists, and nephrologists, is crucial for providing optimal care and improving patient outcomes. Ultimately, hypercalcemia requires a proactive and patient-centered approach, emphasizing not only the resolution of acute symptoms but also the long-term management and prevention of future episodes.

## Conclusions

Hypercalcemia is a multifactorial condition with a wide range of underlying causes, including malignancies, endocrine disorders, and medication use. Due to its diverse etiology, a thorough and systematic diagnostic approach is essential for identifying the underlying cause and determining the appropriate treatment. Despite its relative prevalence, hypercalcemia is frequently undiagnosed because of a lack of clinical suspicion.

Early detection and timely intervention are essential to prevent life-threatening complications, such as renal failure, arrhythmias, and neurologic disturbances. Ongoing monitoring of calcium levels and assessment of underlying conditions are essential for the effective resolution of hypercalcemia and the prevention of recurrence.

Minimizing psychiatric symptoms in hypercalcemia is a significant risk that can compromise both diagnosis and effective treatment of the condition. To ensure that these patients receive proper care, it is crucial that healthcare professionals adopt a comprehensive approach that considers both the psychiatric and metabolic aspects of hypercalcemia. Early recognition, thorough evaluation, and appropriate treatment not only improve clinical outcomes but also prevent long-term complications, leading to a more favorable prognosis for patients.

## References

[1] Walker MD, Shane E (2022). Hypercalcemia: a review. JAMA.

[2] Yamamoto T, Ecarot B, Glorieux FH (1991). In vivo osteogenic activity of isolated human bone cells. J Bone Miner Res.

[3] Rosol T, Capen C (1992). Mechanisms of cancer-induced hypercalcemia. Lab Invest.

[4] Schilling T, Pecherstorfer M, Blind E, Leidig G, Ziegler R, Raue F (1993). Parathyroid hormone-related protein (PTHrP) does not regulate 1,25-dihydroxyvitamin D serum levels in hypercalcemia of malignancy. J Clin Endocrinol Metab.

[5] Carroll M, Schade D (2003). A practical approach to hypercalcemia. Am Fam Physician.

[6] Minisola S, Pepe J, Piemonte S, Cipriani C (2015). The diagnosis and management of hypercalcaemia. BMJ.

[7] Mundy G, Guise T (2005). Hypercalcemia of malignancy: pathogenesis and treatment. J Clin Oncol.

[8] Toschi E, Wolpert H (2016). Utility of continuous glucose monitoring in type 1 and type 2 diabetes. Endocrinol Metab Clin North Am.

[9] Pal L, Cummings S (2009). Hypercalcemia in the elderly. J Clin Endocrinol Metab.

[10] Chaiton M, Schwartz R, Cohen JE, Soule E, Eissenberg T (2018). Association of Ontario's ban on menthol cigarettes with smoking behavior 1 month after implementation. JAMA Intern Med.

[11] Elder GJ (2020). Radiographic absorptiometry: a step in solving the CKD fracture puzzle. Kidney Int.

